# Drivers linking farmers’ decision-making with farm performance: A systematic review and future research agenda

**DOI:** 10.1016/j.heliyon.2023.e20820

**Published:** 2023-10-10

**Authors:** Juan P. Taramuel-Taramuel, Iván Alonso Montoya-Restrepo, Dursun Barrios

**Affiliations:** a Facultad de Ciencias Económicas, Universidad Nacional de Colombia, Bogotá D.C., Colombia; b Facultad de Minas, Universidad Nacional de Colombia, Medellín, Colombia; c Grupo de Investigación Biogénesis, Facultad de Ciencias Agrarias, Universidad Nacional de Colombia, Bogotá D.C., Colombia

**Keywords:** Agribusiness, Farmer behavior, Farming decisions, Farm management, Managerial capacity

## Abstract

A strong aptitude for making sound decisions on a farm is closely linked to favorable farm outcomes, and this finding has been observed across diverse types of farm businesses and geographic locations. Traditionally, research in farm management has addressed the drivers of decision-making and performance as separate entities; however, this article presents novel evidence on the relationship between farmers' decision-making and farm performance. We also examine this association in various contexts of farm decision-making, spanning the past decade. Our comprehensive review encompasses 24 empirical studies conducted in accordance with the preferred reporting items for systematic reviews and meta-analyses (PRISMA) guidelines. The analysis focuses on research topics, performance measures, and methodological perspectives. The findings reveal seven key research topics: farmers' management capacity; the influence of management and farm structure; farmers' emotional attachment to their businesses; personal aspects, farm characteristics, and institutional settings; the significance of farm recordkeeping; joint decisions in farm decision-making processes; and rational inefficiencies in farm decisions. Most studies employed conventional farm performance measures, including financial indicators, technical efficiency, and productivity indicators. Existing studies have predominantly used quantitative methodologies. We also identified research gaps and provide suggestions for future investigations in this field. Our results underscore the pivotal role of decision-making ability in shaping farmers’ managerial capacity and, consequently, farm performance.

## 1 Introduction

Human choice behavior is a mental process that converts perceptions of alternatives into a choice, which can range from an intuitive/impulsive decision to a rational/well-founded one [1]. Typically, the decision-making process of farm managers involves problem framing, judgment, evaluation, and choice; this process is not necessarily linear, as the farmer searches for and processes the available information, and makes the choice based on the selected information in line with his or her experience, motivations, and aspirations/goals [2]. This topic has been relevant in farm management research because it seems be a key factor for understanding why some farmers have better farm results than others operating in similar production conditions [3,4]. In farm businesses, decision-making can shape the organization's success or failure; a high ability to make good decisions is associated with good business results [5]. Understanding how farmers make decisions is crucial for extension professionals in designing effective tailor-made interventions [6], as well as in creating strategies to improve the farm manager's ability [7].

In today's agricultural landscape, farm businesses encounter a multitude of fresh challenges and opportunities arising from a fiercely competitive business environment and a growing emphasis on sustainability [8], as well as opportunities to participate in global markets [9]. At the same time, they must deal with the highly uncertain nature of the agricultural sector, particularly in terms of variations in weather and prices. These factors require that farm managers not only have technical capabilities, but also good farm management decision-making skills that allow them to make the correct decisions to solve problems and take advantage of new opportunities [10]. Al-Rimawi et al. [11] have argued that a poor decision-making process can compromise a farm manager's ability and this, in turn, has consequences on farm performance. A better understanding of how farmers make decisions would be useful in defining successful strategies, policies, and programs aimed at improving their decision-making and, consequently, their managerial capacity [7]. For example, extension agents and technical advisers could take advantage of this information to design farmer-oriented interventions [6,12].

Previous reviews have focused on factors influencing farmers' decision-making as something separate from farm performance. Decisions made by farmers can be related to production, crop management, farm expansion, adoption of new technologies and sustainable farming practices, diversification of agricultural production, risk management, and other farming choices. Some studies have addressed the drivers in farmers' decision-making related to livestock management practices [13,14], agricultural soil management [15], adoption of best management practices [16], or organic farming [17]. The drivers for these decisions include the farmer's socio-demographic factors, goals, motivations, and psychological aspects; farm household characteristics and available technology; biophysical environment; institutional setting; and other determinants [7,18]. Looking at farm performance, Tey and Brindal [19] reviewed factors influencing farm profitability, agricultural productivity [20], or technical efficiency in farm businesses [21]. Farm performance can be explained, in part, in terms of farm characteristics and agency factors including education, age, experience, bookkeeping, having a farm successor, and decision-making ability [22].

Our paper distinguishes itself from previous work on the subject in two ways. First, it provides a comprehensive analysis of empirical evidence regarding the correlation between farmers' decision-making and farm performance. Second, this analysis encompasses various farm businesses, geographical contexts, and covers a span of the last decade. The objective of this review was to investigate the relationship between the drivers of farmers’ decision-making and farm performance. The following research questions are addressed.

1. What are the key research topics in farmers' decision-making and farm performance?
2. How is farm performance measured?
3. What types of samples are included in the studies?
4. What statistical analysis and research methods were employed in the studies?
5. What research gaps and directions for future research appeared in these studies?

The remainder of this article is structured as follows: a brief conceptual framework is presented in the next section. Subsequently, in Section 3, we discuss the method applied in the review of empirical studies of farmers’ decision-making and farm performance. In Section 4, we present the main results and discussion. Section 5 points out directions for future lines of research, and Section 6 concludes.

## 2 Relationship between drivers of farmers’ decision-making and farm business performance

A farmer's decision-making process has three main stages: (a) planning (the determination of the problem and analysis of alternatives); (b) implementation of a course of action; and (c) evaluation/control of deviations from the established plan [4]. Performance in agricultural enterprises is typically understood in terms of technical and economic efficiency (productivity, profitability, etc.). Technical efficiency refers to the ability of a decision-maker to produce a maximum output given a set of inputs and technology, or alternatively, to produce using the least possible quantity of inputs [23]. In terms of economic efficiency, the cost of the inputs is considered, so the objective will be to maximize production using the least amount of inputs that are the most cost-efficient [24]. In this way, a farmer with high management capacity (having good decision-making ability and appropriate personal characteristics/skills) will make optimal use of inputs to produce results that maximize economic returns [25].

**Fig. 1:**
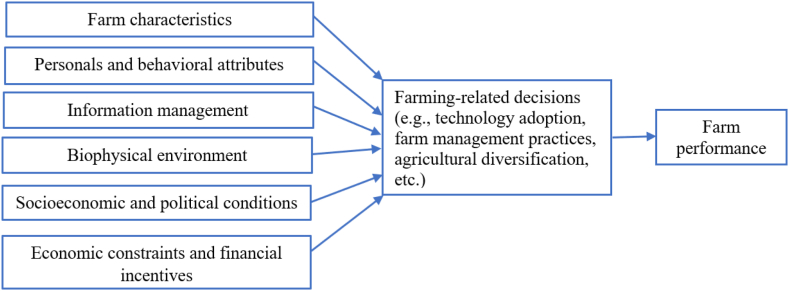
Conceptual framework linking farmers' decision-making to farm performance. *Source:* Own elaboration based on Rougoor et al. [3], Solano et al. [5], Hansson [24], Mäkinen [26], and Bartkowski and Bartke [15].

Fig. 1 illustrates our conceptual framework linking the drivers of a farmer's decision-making with farm performance. It groups the factors involved in this relationship in six categories: farm characteristics, personal and behavioral attributes, information management, biophysical environment, socioeconomic and political conditions, and economic constraints/financial incentives. The main thesis suggested by the framework is that the decision-making process influences farm performance.

In the first factor, farm decisions are influenced by various structural factors. Among these factors, farm size stands out as one of the most widely debated. Larger farms have the advantage of economies of scale and ample space, and their financial capacity often becomes a driving force in making decisions such as adopting new technologies; for example, larger farms tend to embrace precision technologies to a greater extent than smaller farms [27]. There is also evidence showing an inverse relationship between productivity and farm size in emerging economies; Rada et al. [28] found that both small and large farms have higher productivity levels compared to medium-size farms. Another driver in farm-related decisions is farm location. Farms that are distant from markets and agricultural services may have less information access to aid decision-making, and larger distances are negatively associated with the adoption of new agricultural practices [29]. On-farm technologies or farm technological level might facilitate/constrain decisions in farming management. Income dependency on the farm can also drive decisions; farmers who rely solely on farm income and are full-time farmers might spend more time in farming activities, work harder and make better decisions [30].

Personal and psychological variables have been recognized as determinants of a farmer's decision-making. Mäkinen [26] argued that to achieve good farm performance, the farm manager should have a clear vision of the business, engage in planning, and set well-defined objectives. Wilson et al. [31] stated that farmers with business objectives oriented to maximize profits, who are experienced and better educated, and have more information sources for decision-making exhibit more efficient results. Russell and Bewley [6] found that farmers prioritize economic motivations in farm decision-making. From classical economic theory, it is assumed that farmers focus mainly on maximizing economic returns when engaged in decision-making [25]. However, farmers do not always pursue purely economic objectives in their businesses. Some non-financial goals that are important include animal welfare, quality of life, free time, time with their family, and reputation among other farmers [6,32]. Some farmers also prioritize animal welfare at the cost of sacrificing the maximum productivity of their animals, which is called rational inefficiency [33]. In terms of psychological aspects, Saengavut and Jirasatthumb [34] found that a positive attitude towards technology and the perception of ease of use explain the decision to adopt a technology. De Lauwere et al. [35] argued that a farmer's attitudes, beliefs, and perceptions play a crucial role in decision-making about participation in a livestock practice; more specifically, they showed that positive attitudes and behavioral beliefs, as well as fewer perceived problems, related to the practice were linked to higher participation. O'Leary et al. [36] found that a profit-oriented attitude and having a growth mindset were the most predictive drivers of farm business profitability. In the same way, Nuthall and Old [37] highlighted the critical role of intuition in shaping farmer decision-making, noting that many farmers use intuition to make farm-related decisions.

The next factor addresses the role of information in a farmer's decision-making, including sources and management. Solano et al. [38] found that farmers prefer technical advisors and family members as sources of information at different stages of the decision-making process. Russell and Bewley [6] reported that farmers use the advice of specialist consultants as the most important source of information, followed by other farmers, family members, and intuition. Hansson [24] found that farm advisers and other farmers or colleagues were the most important sources of information, which had a positive impact on economic efficiency. Farmers who use reliable and timely information are expected to have better agribusiness performance indicators [39]. Regarding information management in the decision-making process, information helps in detecting and defining problems, but it is necessary to record and evaluate information to select alternatives and control results [24]. Trip et al. [40] found that farm businesses with a high intensity of recording information and a high level of outcome evaluation showed better technical efficiency. Similarly, Manevska-Tasevska and Hansson [41] reported that farmers who track their decision outcomes, prepare budgets, and follow up on records achieved better technical efficiency scores.

The fourth and fifth factors are related to environmental factors. The socioeconomic, institutional, and biophysical environments also play a role in a farmer's decision-making. Some of these elements are out of the farmer's control, including the weather, biological processes, the incidence of pests, or changes in the prices of inputs/products on the market [3]. Climate conditions shape farm production decisions such as planting and harvesting. Institutional setting such as access to credit and extension encourage the decision to adopt agricultural innovations [42]. Additionally, farmers belonging to farmer organizations influence the choice to adopt an agricultural technology [43] or a farming practice [44]. Finally, the sixth factor is related to economic constraints and financial incentives, which have long been recognized as restraints on technology adoption or farm investment decisions. The costs, availability, and access to financing services can constrain/stimulate the decision to adopt a technology [45]. However, the cost of implementation does not always limit the choice [34].

## 3 Methodology

This systematic literature review analyzed empirical studies evaluating the relationship between determinants of a farmer's decision-making and farm performance. The study followed the statements and guidelines of the preferred reporting items for systematic reviews and meta-analyses (PRISMA; [46]). Specifically, we did a qualitative systematic review, and we used the following process: formulation of a research question, development of a pre-specified inclusion criteria, collection of empirical literature, and analysis of data [47].

### 3.1 Inclusion and Exclusion criteria

The inclusion criteria employed in this study are detailed in Table 1. We screened the documents for additional analysis using the Rayyan web application [48].

**Table 1:** Inclusion criteria for the literature review.

Inclusion Criteria
Research on farms, agricultural enterprises, or agribusiness
Studies evaluating the relationship between farm-level decision-making and farm performance (in terms of financial and non-financial outcomes, productivity, efficiency, etc.)
Aspects of decision-making process regarding personal factors (motivations, demographics, psychological), business goals, production planning, shared (or solitary) decisions, records management, sources of information, work with information (processing information), and evaluation and control of the decision outcomes
Empirical research
Written in English

### 3.2 Data sources and search strategies

The literature search was done in March 2023. The search was limited to articles published between January 2013 and December 2022. We focused on the last 10 years because our goal was to analyze the most recent developments. We employed Scopus and Web of Science because these two databases cover a wide range of publishers, have highly reputable indexed journals, and reduce the bias inherent in using only one database. In the search, we used keywords and syntagmas based on the logic of Boolean operators. The research equation was: (TITLE-ABS-KEY (“decision making” OR “decision-making” OR “decision-making process”) AND TITLE (“Farm*” OR “agribusiness” OR “agricult*”) AND TITLE-ABS-KEY (“performance” OR “efficiency” OR “productivity”)). The same search terms were used in both databases.

**Fig. 2:**
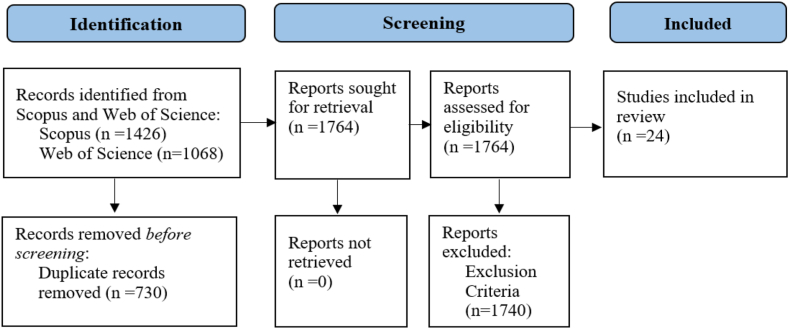
Flow diagram of study selection in the relationship drivers of farmers' decision-making and farm performance. *Source:* The authors.

Fig. 2 present a flow diagram of the study selection procedures, which were as follows: 1426 documents were identified from Scopus and 1068 documents from Web of Science; 1764 documents remained after removal of the 730 duplicates; 1740 documents were excluded based on the inclusion criteria. This left 24 articles that were selected for the qualitative analysis. We used the Rayyan program to eliminate duplicates, as well as to screen the articles [48]. We then employed Power BI to visualize the geographical distribution of the reviewed papers. Additionally, we used the VOSviewer tool to analyze the co-occurrence of words [49]. Finally, we categorized the empirical articles looking for common patterns in research topics, farm performance indicators, and methodological approaches.

## 4 Results and discussion

### 4.1 Overview of the primary articles

**Fig. 3:**
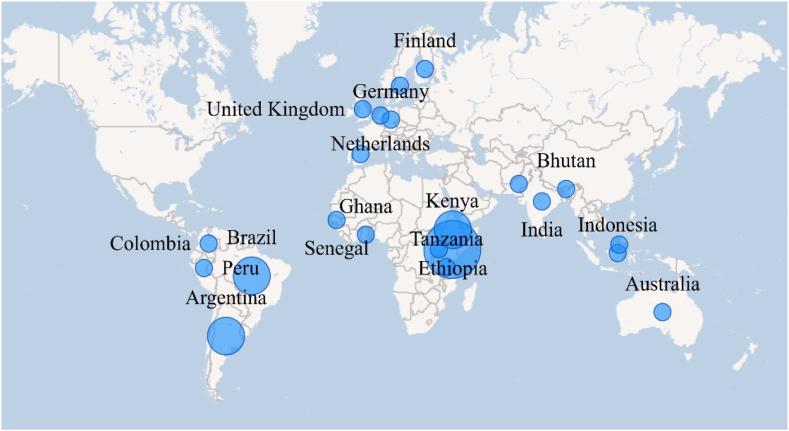
Geographical distribution of studies on the relationship drivers of farmers' decision-making and farm performance. *Source:* The authors using Power BI.

Based on the 24 documents selected, we identified a growing interest in addressing the relationship between farmers’ decision-making and farm performance in the academic setting. The publishing ratios have been increasing, as follows: 2013–2017 (7 documents), 2018–2020 (8 documents), and 2021–2022 (9 documents). Most of the reviewed studies were conducted in Africa (33.3 %), followed by South America and Europe (each 22.2 %), South and Southeast Asia (18.5 %), and Australia (3.7 %). Fig. 3 shows the geographical distribution of the reviewed studies.

It was surprising that much of the reviewed research was focused on low-middle-income countries; a plausible explanation could be related to the crucial role of the agriculture sector in economic development, especially in those countries with a high contribution of this sector to GDP growth, so substantial research efforts are growing to find ways to improve farm-level efficiency [50]. It does not mean that farmer's decision-making in developed economies is a waning research topic, for example, Emirhüseyinoğlu and Ryan [51] studied how farming decisions shape profitability, by using simulation models they explored how to maximize farm profits, while also considering sustainability goals. Research may focus on other concerns, including understanding decisions in environmental considerations from farm management practices [14,52], adopting sustainable agricultural production systems [53], or analyzing financial and non-financial influencing factors on the strategic farm expansion decision-making process [54]. Farmer behavior is also a relevant research topic in other geographic contexts, including in China. Shang and Xiong [55] explored how psychological and sociodemographic determinants explain decisions on implementing farm risk management strategies. In the same way, Hou and Hou [56] employed a psychological model to understand the adoption decision of sustainable agricultural production. In sum, while much attention regarding the relationship drivers of farmer's decisions and farm performance has been directed toward low-middle-income countries, farmer behavior research in developed economies continues to thrive in other concerns including environmental sustainability.

**Fig. 4:**
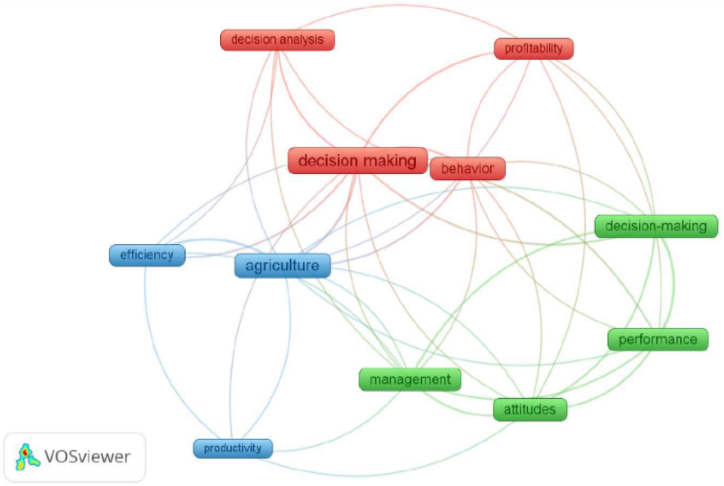
Word network visualization in the relationship drivers of farmers' decision-making and farm performance. *Source:* The authors using VOSviewer.

In the co-word analysis of keywords, we identified three clusters in the word network, as shown in Fig. 4: Cluster 1 is represented in red, and comprises four items (decision-making, decision analysis, behavior, and profitability); Cluster 2 is represented in green and comprises four items (decision-making, performance, attitudes, and management); and Cluster 3 is represented in blue and comprises three items (agriculture, efficiency, and productivity). It is evident that decision-making is linked to farm performance (e.g., profitability) in a direct way and mediated by the word “agriculture” to efficiency and productivity.

### 4.2 Research topics

We identified seven research topics, which are shown in Table 2. The first, the *farmer's management capacity*, seeks to understand how personal attributes and aspects of decision-making process shape farm performance [3]. For example, Mäkinen [26] studied how the farmer's managerial thinking and effectiveness of management process explain farm financial performance, Carrer et al. [57] analyzed the role of the farmer's expectations and adoption of farm management information systems on technical efficiency, and Fadzim et al. [30] studied demographic and socioeconomic factors affecting farm efficiency. The second topic is *the role of management and farm structure*. In addition to studying management capacity, this research topic also considers farm size, diversification, production system, intensification, and other farm characteristics [58], as well as the technological level of the farm [59,60]. The third topic, *farmers' emotional attachment to their business*, addresses how non-economic factors, including socioemotional wealth, influence the decision to remain in the business even when returns are minimal [32]. The fourth topic, *personal aspects, farm characteristics, and institutional settings*, covers institutional, farm characteristics, social, and market drivers. For example, Bravo-Monroy et al. [44] found that membership in farmer organizations, population density, years of formal schooling, agricultural education, and the role of institutions in terms of technical assistance or satisfaction with institutional incentives influence a farmer's decision-making. The farmer's attitudes, preferences, and perceptions are also important in understanding their decision-making [12,61]. The profit-seeking goal is one of the main drivers of farm decisions [62], and farmers prefer the most profitable crops [63].

**Table 2:** Research topics related to farmers’ decision-making and farm performance.

Topic	Purposes	Main findings	Sources
**Farmers' management capacity**	This research topic focuses on (a) demographic characteristics including age, experience, education, and farming goals, and (b) use of information in the human decision-making process.	• Clear business goals and plans are linked to better farm results. • Planning and monitoring of production using recordkeeping tools are related to farm performance. However, it is not a conclusive finding. • Experienced and full-time farmers tend to have better performance indicators.	[26] [57] [30]
**The role of management and farm structure**	The objective of this topic is to understand how (a) decision-making, (b) personal attributes, and (c) farm characteristics shape farm outcomes.	• The use of external information and farm records, as well as setting clear objectives, planning, and maintaining control have a positive impact on farm results. • Larger farms show better outcomes. • Married and more educated farmers with a primary source of income from farms have higher farm technical efficiency. • Management activities including farm recordkeeping, production planning, and using records in decision-making are associated with farm performance.	[58] [59] [60]
**Farmers' emotional attachment to their business**	This research topic seeks to better understand o farmers' goals, motivations, and the role of emotional attachment (feelings and emotions) on farm survival.	• Farms were able to remain in the business despite minimal economic returns by engaging in four different strategic behaviors: diversifying the business, increasing levels of debt, sacrificing family time and activities, and accepting less than optimal business performance. • Emotional attachment plays a critical role in making decisions about the business.	[32]
**Personal aspects, farm characteristics, and institutional settings**	This research topic covers (a) farmers' beliefs, attitudes, perceptions, goals, and demographic characteristics; (b) farm characteristics including size, technology availability, farm production practices, livestock and cropping systems, and biophysical conditions; and (c) institutional environment, including the role of institutions, membership of collectives, access to technical assistance and credit, and market distance.	• The social identity and relations of production are the main drivers explaining the farmers' decision-making process. • Recordkeeping is an important tool to make decisions. • It is important to understand farmers' attitudes, perceptions, and preferences in their decision-making to design tailor-made interventions. • Higher farm productivity is linked to improved environmental performance. • Profit-seeking is one of the main goals of farmers. These farmers are willing to bear additional risks to improve farm results. • Government support (credits, programs, and subsidies) influences farmers' decisions. • Access to extension and credit services shape farmers' decisions. • As expected, farmers who decide to adopt technology and production practices including mobile internet, new crop varieties, improved farming practices, and farm management tools show better farm income and outcomes. • Farmer organizations (collectives, cooperatives, unions, etc.) are an important source of information. • Organic agriculture seems to be slightly more technically efficient than conventional farming.	[44] [62] [61] [29] [12] [64] [65] [63] [66] [67] [68]
**The role of farm recordkeeping**	This research topic targets the role of farm recordkeeping, including information management and farm benchmarking, in the process of decision-making.	• Farmers who engaged in some form of comparative recordkeeping activity were more open to farm management changes to improve farm performance.	[69] [70]
		• Good farm record management allows assessing farm performance in an accurate way.	
		• It is crucial to encourage maintaining farm records to support decision-making.	
**Joint decisions in farm decision-making processes**	The objective of this research topic is to address joint or solo farm decision-making considering personal aspects and farm characteristics.	• It seems that there is a monopolization of farm decisions by male decision-makers. However, joint decisions are a frequent decision-making approach.	[71] [72] [73]
		• Joint and female decision-makers may have an adverse effect on farm productivity and agricultural marketing outcomes.	
		• There is an important involvement of women in farm decision-making and in physical farm operations.	
**The rational inefficiency in farm decisions**	This research topic's objective is to test the rational inefficiency hypothesis, specifically when farmers improve animal welfare at the expense of efficiency.	• It is important to consider that some farmers might prefer animal welfare instead of pushing their animals towards their maximal productivity. In other words, inefficiency in production results from rational production decisions made by the farmers.	[33]

The fifth topic, *the role of farm recordkeeping*, considers farm recordkeeping and management information, which are useful tools to make informed decisions because they help to define problems and control farm outcomes [69,70]. The sixth research topic covers *joint decisions in farm decision-making processes*. Despite a monopolization of farm decisions by one decision-maker being a common decision-making approach, some tactical and strategic decisions are shared or delegated to farm staff, family members, and technical advisors [74]. In this research topic, some studies are related to differences in marketing efficiency for male, female, and joint decision-making farming households [71]; differences in the inverse relationship (farm size and productivity) in joint (husband and spouse) and individual farming decision-making [72]; and the involvement of women in farm decisions and operations [73]. An inconclusive finding is that joint decisions might be related to adverse farm outcomes, but preliminary explanations are related to gender-based discrimination environment, as well as to miscommunication and missing information in joint decisions [71,72]. Finally, the seventh topic, *rational inefficiency in farm decisions*, is focused on understanding farm decisions in which farmers do not maximize outcomes, but instead prefer consider animal welfare or environmental concerns [33].

### 4.3 Farm performance measures

Farm business performance can be conceptualized and measured from different approaches, as shown in Table 3. Recent methods evaluate performance in a holistic way, where non-economic measures including environmental and farmer welfare indicators are considered alongside the traditional measures. Convectional approaches include financial, productivity, and technical efficiency. For example, Effendy et al. [66] employed total factor productivity (TFP) as a performance indicator when they analyzed the role of socioeconomic factors on the adoption of agricultural innovations. Additionally, business farm performance can be evaluated by self-reported measures including satisfaction with performance, which can be useful in developing countries, where farmers usually do not keep farm records [70] or when managers are reluctant to disclosure sensitive financial performance information [75].

**Table 3:** Measuring business farm performance.

Category	Indicators	Sources
Financial	Operating margin (%), profitability ratio, and profit ($)	[26]
	Profit (farm viability)	[58]
	Profitability (%) and other financial outcomes, farm income, milk yield, and other reproductive/productive indicators	[59]
Productivity-related indicators	Milk yield (milk per cow/year)	[61]
	Productivity (tons/ha) and total factor productivity (TFP)	[66]
	Wheat yield (kg/ha)	[68]
	Productivity (t/ha)	[73]
Technical efficiency	Stochastic frontier of production analysis (SFA)	[57]
	Data envelopment analysis (DEA) method	[30]
	Multidirectional efficiency analysis (MEA) and DEA approach, milk, and meat output (total revenue), other revenue sources	[33]
	DEA method	[60]
	Cobb–Douglas stochastic frontier model	[67]
	Cobb-Douglas production function, and productivity (kg/ha)	[72]
Performance using quasi-objective and subjective measures	Self-rated productivity and profitability	[69]
	Estimated yield milk (by the farmer), farm income (from crop and milk sales)	[29]
	Self-rated profit margin, profit and loss, and productive indicators	[70]
	Self-reported farm milk yield (L/day)	[65]
Mixed-measures and others	Farm survival	[32]
	Productivity (Kg/ha), profitability (%), production costs, income from farming	[44]
	On-farm and off-farm income	[62]
	Farm income, reproductive indicator (delivered piglets per sow), total revenue, and production costs	[12]
	Profit ($/ha), labor productivity (kg grain/$labor), grain yield (kg/ha), environmental performance: water productivity (kg grain/water-hour), nitrogen use efficiency (kg grain/kg N), phosphorus use efficiency (kg grain/kg P), pesticide use efficiency (0–75 points), and greenhouse gas emissions (kg grain/kg CO_2_ equiv.)	[64]
	Productivity (t/ha), operating profit ($/ha), production costs ($/ha); environmental performance: farm nitrogen balance (kg/ha); non-economic performance: household leisure time (h/year)	[63]
	Agricultural marketing efficiency: producers' share in market price (%), marketing costs ($), gross marketing margin ($), net marketing margin ($)	[71]

### 4.4 Research approaches and analysis techniques

Table 4 summarizes the methodological approaches evaluating the relationship between drivers of farmers’ decision-making and farm performance. The systematic analysis identified quantitative approaches as the most common research method, followed by mixed-methods, and qualitative approaches. For quantitative studies, the minimum sample size was 40 farm businesses and a maximum of 13,095 agribusinesses chosen using both probability and non-probability sampling. The most common instrument for collecting information was the survey and different correlational analyses were used.

**Table 4:** Methodological approaches to farmers’ decision-making and farm performance.

Approach	Sample size; sampling method; research sample (agribusiness)	Instrument	Analysis Technique	Sources
**Quantitative**	117; non-probability sampling (voluntary participation); dairy	Farm Accountancy Data Network (FADN) survey, financial statements	Structural equation modeling (SEM)	[26]
	57; stratified random sampling; dairy	Survey and on-farm observation	Logistic regression model	[58]
	98; simple random sampling; citrus	Structured questionnaire	Stochastic production frontier translog	[57]
	375; cluster sampling; cocoa	Structured questionnaire	Tobit model	[30]
	109 (simple random sampling), 40 (non-probability sampling); horticultural	Survey (questionnaire)	Propensity score matching (PSM) technique	[62]
	60; non-probability sampling; dairy	Survey (questionnaire)	Kruskal–Wallis one-way test	[61]
	421; non-probability sampling; dairy	FADN survey	MEA & DEA scores, Chi-square tests, *t*-tests	[33]
	157; simple random sampling; sheep	Survey (questionnaire)	Canonical correlation models (CCA)	[59]
	13,095; non-probability sampling; dairy	Survey (questionnaire)	Chi-square tests, *t*-tests, logistic regression model, factor analysis	[29]
	118; simple random sampling; dairy	Survey (questionnaire)	Ordinal logistic regression	[70]
	79 (panel data), 55 (follow-up); non-probability sampling; pork	FADN survey, follow-up survey	The fixed effects model (panel data), multivariable linear regression analyses (follow-up)	[12]
	378; non-probability sampling; dairy	Survey (questionnaire)	Cluster analysis (K-means)	[65]
	40; stratified random sampling; bees	Survey (questionnaire)	Tobit model	[60]
	329; stratified random sampling; rice	Survey (questionnaire)	Double-hurdle model	[66]
	400; multi-stage sampling (purposive, probability proportion and stratified); vegetables	Survey (questionnaire)	Stochastic frontier model, Probit model	[67]
	460; multi-stage sampling (purposive and probability proportion); wheat	Survey (questionnaire)	PSM technique (logit model, covariate balance test, sensitivity analysis)	[68]
	1931; multi-stage sampling (random, proportional); rice	Survey (questionnaire)	Cobb-Douglas production function, multivariable linear regression analyses	[72]
	240; purposive sampling; potato	Survey (questionnaire)	Spearman's correlation, Kruskal–Wallis one-way test, Mann–Whitney *U* test	[73]
**Mixed-Methods**	Qualitative: 14 farmers. Quantitative: 134 farmers; non-probability sampling; coffee	Qualitative: first-hand observations, in-depth interviews, informal dialogues; quantitative: survey (questionnaire)	Chi-square tests, Kruskal–Wallis test, Classification and Regression Trees (CART) analysis	[44]
	Qualitative: 2 focus groups (45 farmers). Quantitative: 65 farmers; snowball sampling; rice	Qualitative: focus group; quantitative: survey (questionnaire)	Linear mixed effect model, ANOVA	[64]
	Qualitative: 20 farmers (rapid system analysis); simple random sampling, 4 farmers (detail system analysis); purposive sampling. Quantitative: modeling; rice and vegetables	Qualitative and quantitative: interviews (structural and semi-structural component)	Farming system analysis, multi-objective optimization model (FarmDESIGN)	[63]
	Qualitative: key informants (12 agricultural experts, 16 community elders, 8 maize collectors, 16 retailers, and 4 wholesalers), 3 focus groups (6–8 farmers in each group). Quantitative: 560 farmers; multi-stage sampling (proportional, purposive); maize	Qualitative: key informant interviews, focus group; quantitative: survey (questionnaire)	Test statistics	[71]
**Qualitative**	20 farms (48 interviews); purposive sampling; dairy	Interviews	Cross-case analysis	[32]
	24 farms; purposive sampling; sheep and beef	Interviews	Inductive approach	[69]

In the mixed approaches, both qualitative and quantitative tools were used. White et al. [64] employed this integrated approach to enable a better understanding of a farmer's perceptions and drivers of farm performance. For qualitative methods, small samples and non-probability sampling were used and instruments involved first-hand observations, interviews, focus groups, and key informants. In the quantitative part, correlation analysis was employed to evaluate the influence between the drivers of a farmer's decision-making.

The qualitative sought to gain a deep understanding of farmers’ decision-making; for that purpose, in-depth interviews were used, and some analysis techniques included cross-case and inductive studies. In terms of type of farm businesses, 50 % of the research samples were livestock farms, and the most common business was the dairy farm. The remaining 50 % correspond to crops and tropical fruits including rice, coffee, wheat, potato, citrus, cocoa, and vegetables.

## 5 Directions for future research

This systematic review centered on three key perspectives concerning the relationship between the drivers of farmers’ decision-making and farm performance: research topics, farm performance measures, and methodological approaches. Through a comprehensive analysis of 24 articles, we identified certain research gaps that warrant further attention. To provide a concise overview, Table 5 presents a summary of potential research directions for future investigations. This paper focused on identifying some aspects of decision-making linking farm performance. The next research step should be aimed at working on alternative strategies for farm business, Kay et al. [76] provide four general strategies: (a) low volume, high-value producers, (b) high volume, low-margin producers, (c) specialty product and service providers, and (d) part-time farmers [10]. For example, Glover and Reay [32] found that farm businesses were able to sustain business despite minimal economic returns by engaging in four different strategic decisions: business diversification, debt raising, prioritization (sacrificing family activities and time), and suboptimal business performance (to keep family relationships).

**Table 5:** Agenda for future studies in farmers’ decision-making and farm performance.

Perspective	Identified gaps	Suggestions for future research
**Topics**	• Compared to demographic factors, economic constraints, and farm characteristics, little focus has been placed on non-economic drivers in farmers' decision-making.	• Future studies should include non-economic factors, such as the role of emotional attachment in farm decisions.
	• There is increasing evidence showing the crucial role of the institutional environment in farmer behavior, particularly in emerging economies. However, it is necessary to gain more understanding of this topic.	• It is suggested that researchers study the role of the institutional setting, including extension and financial services, farmer organizations, and government programs shaping agricultural decisions and farm performance. It would be useful to enhance tailor-made interventions.
	• In developing countries where farmers usually do not keep track of what has happened on their farms, it is crucial to understand why they do not keep farm records.	• More studies on the importance of farm records and benchmarking to design tools and strategies aiding farmer's decisions.
	• The role of gender in farm decision-making is not fully understood.	• It would be of great interest to expand women's role in farm decision-making and farm performance, as for example, some research has shown that joint and female farming decisions may lead to poor farm results.
	• While productivity and technical efficiency have been the dominant approaches for farm performance, this article calls for more research on more compressive farm performance measurements including social and environmental indicators. Likewise, more research more on rational inefficiency would be desirable, particularly in trade-offs of animal welfare and environmental considerations.	• Future studies should focus on farm performance in terms of social and sustainability-related aspects, such as a better understanding of changing farming practices, including organic production.
**Sample types and locations**	• Despite there being research on livestock farming and some crops, there is a dearth of research on various tropical fruits such as avocado.	• A better understanding of agricultural systems for different livestock and crops would be very useful because of the heterogeneous nature of farming systems. Considering countries have different stage of economic development and socioeconomic, biophysical, and institutional environments, drivers of farming decision-making would also be different.
**Methodologies**	• Little focus has been placed on longitudinal studies; most research has worked on cross-sectional data.	• It is suggested that researchers analyze farmers' decision-making over a period, because it may make time to have an impact on farm performance.
	• There is a dearth of research evaluating the determinants of farmer behavior using experimental approaches.	• Future studies should focus on controlled studies to capture actual drivers in farmer behavior.
	• Relatively less empirical attention has been paid to overcoming the lack of performance data, particularly in developing nations.	• It would be of great interest to use alternative ways to measure farm performance including perceived performance, because farmers typically do not have farm records in developing countries, or they are reluctant to reveal sensitive performance information.
	• Little focus has been placed on implementing qualitative and mixed approaches.	• Qualitative research including ethnographic techniques would be useful to better understand farmer behavior.

### 5.1 Limitations

Considering that we limited our review to Scopus and Web of Science databases, some studies may have been excluded from the review. Some documents may have been accidently omitted due to human error. Future reviews might also cover a longer period to look for more insights into the relationship between farmers’ decision-making and farm performance.

## 6 Conclusions

The relationship between the drivers of farmers’ decision-making and farm performance is an area of increasing research in the field of farm management, especially when focused on economically developing countries or emerging economies. In this paper, we focused on farm decisions linked to farm performance addressing the research topics, performance indicators, and methodological approaches. We found that factors influencing farmer decision-making behaviors can be grouped by (a) farm characteristics, (b) personal and behavioral attributes, (c) information management, (d) biophysical environment, (e) socioeconomic and political conditions, and (f) economic constraints/financial incentives. Economic-related motivations appear to be the main determinants of farmer choices, however, non-economic factors including emotional attachment might help to clarify the relationship. Some topics that future studies should address include the role of women in farm decisions and performance, non-conventional farm performance measures, and how institutional and geographical contexts shape farmer behavior. A better understanding of how farmers make decisions, the determinates, and the role of decision-making in farm performance would be useful in designing effective public and private interventions, for example, by allow agricultural extension agents to design tailored-made programs. Additionally, this paper grounds further research on developing strategies for agribusinesses. In a practical sense, if farmers are aware that the way they make their decisions may explain inadequate farm performance; they can work on improving their decision-making to achieve better farm outcomes.

## Funding statement

This work was partially supported by 10.13039/501100002753Universidad Nacional de Colombia and the Biogénesis research group.

## Data availability statement

Data associated with this study are available online through the Scopus and Web of Science databases.

No additional information is available for this paper.

## CRediT authorship contribution statement

**Juan P. Taramuel-Taramuel:** Conceptualization, Data curation, Formal analysis, Investigation, Methodology, Visualization, Writing – original draft. **Iván Alonso Montoya-Restrepo:** Formal analysis, Investigation, Supervision, Validation, Writing – review & editing. **Dursun Barrios:** Formal analysis, Funding acquisition, Methodology, Project administration, Resources, Software, Supervision, Writing – review & editing.

## Declaration of competing interest

The authors declare that they have no known competing financial interests or personal relationships that could have appeared to influence the work reported in this paper.
